# Egg-trading worms start reciprocation with caution, respond with confidence and care about partners’ quality

**DOI:** 10.1038/s41598-021-89979-7

**Published:** 2021-05-18

**Authors:** Maria Cristina Lorenzi, Dáša Schleicherová, Franco G. Robles-Guerrero, Michela Dumas, Alice Araguas

**Affiliations:** 1 Laboratoire d’Ethologie Expérimentale et Comparée, Université Sorbonne Paris Nord, 99 Avenue J.-B. Clément, 93430 Villetaneuse, France; 2 Dipartimento di Scienze della Vita e Biologia dei Sistemi, Università di Torino, Via Accademia Albertina 13, 10123 Torino, Italy

**Keywords:** Evolution, Social evolution

## Abstract

Conditional reciprocity (help someone who helped you before) explains the evolution of cooperation among unrelated individuals who take turns helping each other. Reciprocity is vulnerable to exploitations, and players are expected to identify uncooperative partners who do not return the help they received. We tested this prediction in the simultaneously hermaphroditic worm, *Ophryotrocha diadema,* which engages in mutual egg donations by alternating sexual roles (one worm releases’ eggs and the other fertilizes them). We set up dyads with different cooperativeness expectations; partners were either the same or a different body size (body size predicts clutch size). Large worms offered larger clutches and did so sooner when paired with large rather than small partners. They also released smaller egg clutches when they *started* egg donations than when they *responded* to a partners’ donation, fulfilling the prediction that a players’ first move will be prudent. Finally, behavioral bodily interactions were more frequent between more size-dissimilar worms, suggesting that worms engaged in low-cost behavioral exchanges before investing in such costly moves as egg donations. These results support the hypothesis that simultaneously hermaphroditic worms follow a conditional reciprocity paradigm and solve the conflict over sexual roles by sharing the costs of reproduction via the male and the female functions.

## Introduction

The evolution of cooperation and altruism by natural selection is one of the most intriguing questions in evolutionary biology: how does natural selection favor the evolution and spreading of behaviors that benefit recipients at a cost to the actors?

Reciprocal altruism, a theory first proposed by Trivers^1^ and later by Axelrod and Hamilton^2^ explains the evolution of cooperation between genetically unrelated individuals. Trivers proposed that even though an animal that spends resources for another may incur a cost at first, once the partner reciprocates, benefits and costs are evenly shared between the two cooperating partners (correlated payoffs^3^). In the long term, if the overall benefits are larger than the costs, they will yield higher fitness to the individuals expressing mutually cooperative behaviors^1^.

Although initially addressed as a mechanism that primarily—or exclusively—explained human behavior because it was considered cognitively demanding^4–7^, reciprocity has been widely reported among animals^3,8^ and cognitive limitations are not currently seen as an obstacle to reciprocation^9,10^.

One well known example of non-human reciprocation occurs among vampire bats, who share food with starving social partners (i.e., regurgitate part of their blood meals). However, they do so not only with related individuals, but also with other members of the colony. In fact, bats are likely to donate food to an unrelated social partner if they received food from that social partner in the past^11^. Intriguingly, the amount of food received predicts the amount of food returned^12,13^. This prediction, possibly difficult to test in bats, was tested in laboratory Norway rats by training them to exchange pieces of food. Rats that donated a valued banana treat were more likely to receive food in return, and to get it sooner, than rats that had donated the less preferred carrot treats^14^. In both the bat and rat examples, the quality of the partner’s donation is central to reciprocal exchanges. Indeed, while reciprocal interactions were initially modelled as discrete and all-or-nothing moves (players could either cooperate or defect), subsequent models have incorporated scaled donations (i.e., variable degrees of cooperation^15,16^).

Reciprocal exchanges have also been described in the context of mating. Mating typically highlights the diverging interests of females and males (sexual conflict^17–19^) evolutionarily ascribed to the unequal investment in size and number of gametes by females and males (anisogamy^20^). Sexual conflict also occurs among hermaphrodites, where it includes the premating conflict over sexual roles, a form of sexual conflict exclusive to hermaphrodites^21^. Typically, in simultaneous hermaphrodites which mate unilaterally, both partners have male and female gametes and can mate in either sex. However, they express only one sexual role at a time and mate either as males or as females at any given mating^22^, meaning if one partner plays female, the other plays male. In fact, a conflict arises if mating in one sex is preferred over mating in the opposite sex (i.e., if the cost/benefit ratio for mating via the female *vs* the male function differs) and if partners share the preference for the same role^23,24^; “the hermaphrodite’s dilemma”^25–27^.

Some hermaphroditic species have channeled the conflict over sexual role decision in a mutually beneficial outcome which consists in alternatingly assuming the female and the male role in successive mating rounds. For instance, individual A plays male at the first mating and female at the next (and concurrently its partner B plays female at the first mating and male at the next). Typically, such hermaphroditic partners keep switching between sexual roles over repeated mating rounds^28–30^, so that in the long-term partners will play female as often as they play male. The alternation of sexual roles has been reported in diverse hermaphroditic organisms, such as fish^31–33^ and annelid worms^34,35^.

If the alternation between sexual roles is contingent on the partner’s behavior (i.e., partner returns the donation received previously), one of the requisites for conditional reciprocity is fulfilled^1,2^. In hermaphroditic worms, experimental data and agent-based simulations do support this hypothesis; hermaphroditic worms take turns donating eggs to partners that have previously donated egg clutches^36^.

Reciprocation is often reported as an evolutionarily stable strategy if players pursue rules such as “help someone who has helped you before” (direct reciprocity^3^), “copy partner’s response”^37^ and the “mirror rule”^38^. Therefore, the decision to perform an action which is costly to the actor and beneficial to another individual is based on the expectation that, judging from past interactions, the individual receiving help will pay it back in the future. So, what about the start of reciprocal exchanges?

The tit-for-tat strategy for the reiterated Prisoner’s dilemma predicts that players start by being cooperative^2,16^. However, whenever the exchanges are not concurrent, the player performing the first move cannot judge the partner from past interactions and is therefore exposed to the sucker’s payoff (i.e., paying the cost without getting any gain in the future). So, if the partner defects rather than returns the help, its investment will not be rewarded^1^; defection is indeed the most rewarding move if players meet only once^2^. Players can weigh their decision to start with a cooperative move based on the costs of the donation, the benefits their partners receive and the probability of getting benefits back in the future, as would be the case for any move in reciprocal exchanges^3^, except that at the first move the evaluation is based on expectations rather than on donations received. To maximize their potential gains, minimize costs and avoid the sucker’s payoff, individuals have to assess whether their partner would be likely to return the help and the quality of help their partner would offer. Theoretical models predict that reciprocity is more likely to emerge when interacting individuals have positively correlated phenotypes^38^.

We investigated the mechanisms of reciprocity in dyads where partners differed by quality, so that expectations of reciprocation differed; high-quality partners would be able to return high-quality donations whereas poor-quality partners would not. We first compared the start and the maintenance of reciprocal exchanges in these dyads. We then tested whether individuals interacted prior to starting reciprocal exchanges and whether the frequency of behavioral interactions increased with increasing quality difference between partners. Finally, we tested whether high-quality partners were preferred to low-quality ones.

We used the hermaphroditic marine polychaete worm *Ophryotrocha diadema* as the study model. These long-lived worms live in sparse populations^39^. This implies low encounter rates and explains why, once two partners meet, they engage in long-term monogamy^40^. Worms reach full sexual maturity on both male and female functions at a body size of ~ 13 segments^41^. Before that, they pass a 40-days-long protandrous phase during which they produce viable sperm and are able to fertilize the eggs of mature hermaphrodites. However, they cannot produce eggs during this stage^42^ making them poor reciprocators. In pairs, fully sexually mature worms typically court each other for days before engaging in pairwise, mutual and sequential exchanges of eggs, because each worm needs the partner’s sperm to fertilize its own eggs (obligately outcrossing simultaneous hermaphrodites)^34^.

Each worm lays an egg clutch every fourth day^34,36^ and up to 12–18 clutches during the first two months of its reproductive life^43^. Producing eggs typically requires a larger investment than producing sperm^44–46^. In *O. diadema* worms, eggs are huge compared to sperm (eggs: 310.46 ± 9.79 μm^47^; sperm: ~ 5 μm^48^) and are released enclosed in a large jelly cocoon, in clutches of ~ 25 eggs^34,39^. Overall, worms invest up to 38% of their bodily resources into a single egg clutch^47^, whereas the investment in sperm is negligible^49^. This makes it plausible that egg laying is the costly move and fertilizing eggs is the cheap one in egg trading. In the reciprocity vocabulary, donating eggs is the cooperative move; refraining from doing so—i.e., doing nothing^15^—is defecting. When a worm donates a clutch of eggs to its partner, it will get eggs back to fertilize after a typical delay of two days^34,36^. Therefore, the time lag between two successive matings makes egg return uncertain and the evaluation of partner propensity to reciprocate eggs is accordingly crucial.

Once the mating pair has formed, worms engage in activities at and outside the nest (e.g., parental care of egg-cocoons, rubbing their partner and foraging, respectively) and regularly make their way back to the nest using the dense web of mucous trails they build^50^. While rubbing takes most of the time partners spend at the nest, mating (egg releasing by one worm and egg fertilizing by the other) is rapid and occurs every 2 days^51^. There is no evidence that worms recognize their mating partners, but they might use chemical cues in the mucous trails and around the nest to ensure that they encounter their partner again^52^.

These worms perceive and process crucial social information enabling them to adjust their sex allocation to current mating opportunity. They invest large amount of resources into the female function (egg production and parental care) when they are kept in isolated pairs (only one partner, low mating opportunities), whereas they strongly diminish their female investment in favor of the male function (motility and aggressive competition for mating, and to a smaller extent, sperm number) when multiple partners and more mating opportunities are available^30,40,49,53–55^. In this condition, established pairs can divorce and mature worms can withhold eggs and play the male role only for weeks or even their whole life^40,56^.

If reciprocity explains mutual egg exchange in paired hermaphrodites (including conditioning egg release to egg release by the partner and matching partner’s clutch size, see^36^), we expect that worms are reluctant to start reciprocal egg exchanges with poor quality partners. Clutch size positively correlates with body size^36,41^ and large partners are usually preferred to small ones^34^; therefore, we used body size as a measure of partner quality. We tested the reciprocity-based hypothesis against the alternative hypothesis that mutual egg exchanges are explained in terms of pseudo-reciprocity, whereby egg donation is primarily beneficial to the egg-releasing worms and only incidentally to their partners. Under a pseudo-reciprocity paradigm, worms are expected to make similar reproductive decisions irrespective of their partner’s quality.

Based on theoretical expectations that partners with correlated phenotypes are more likely to engage in reciprocity^38^, we predicted that dyads of size-matched partners would start mutual egg exchanges sooner than size-unmatched dyads. Under the hypothesis that reciprocal relationships are built through increasing investment^15^, we also predicted that worms would make prudent decisions—and donate smaller egg clutches—when they made the first rather than the second move (i.e., when they start egg donation *vs* when they respond to partners’ egg donation). Next, following recent emphasis on the practice of “testing the waters” prior to investing in reciprocity^13,15,57^, we predicted that individuals would engage in behavioral interactions before starting egg exchanges and would do so more extensively prior to starting egg exchanges when paired with partners of dissimilar sizes. Finally, we tested worm mate-preference based on body size by measuring whether focal worms preferentially engaged in courting the larger partner when offered the choice between two partners of dissimilar size.

## Results

### Experiment 1: reciprocation in matched and unmatched dyads

**Figure 1:**
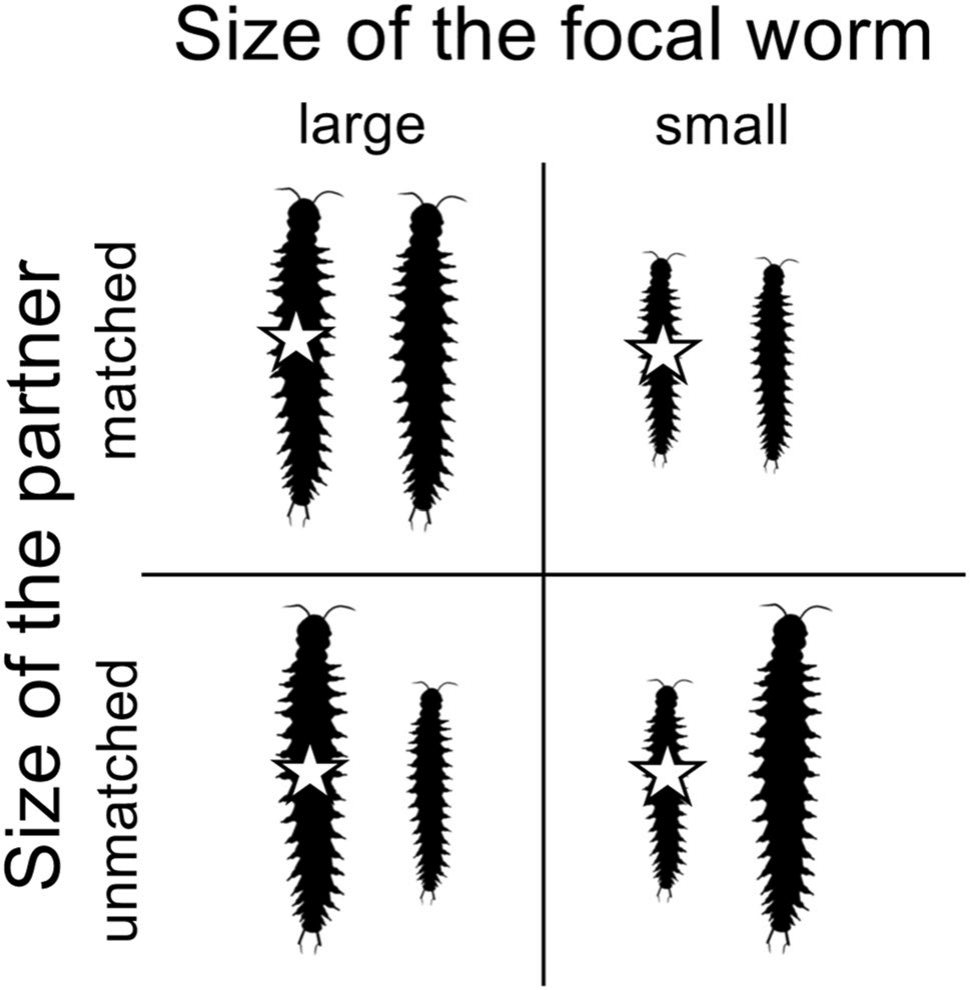
Schematic representation of the experimental design for Experiment 1—reciprocation in size-matched and size-unmatched dyads. The effect of the size of the focal worm (large/small) and that of the partner (matched/unmatched) were tested. The star indicates the focal worms (i.e., yellow-phenotype worms, see Materials and Methods) used to take measurements (number of replicates: 36 per condition). Worm silhouettes by Scott Hartman are available under Public Domain license at PhyloPic (http://phylopic.org/).

During the three-week experiment where large or small focal worms were paired with either size-matched or size-unmatched partners (Fig. 1), the large focal worms exchanged a total of 455 cocoons (and 9539 eggs) with their large or small partners; the small focal worms exchanged 400 cocoons (and 8125 eggs) with their own (large or small) partners.

#### Latency to start egg donation

**Figure 2:**
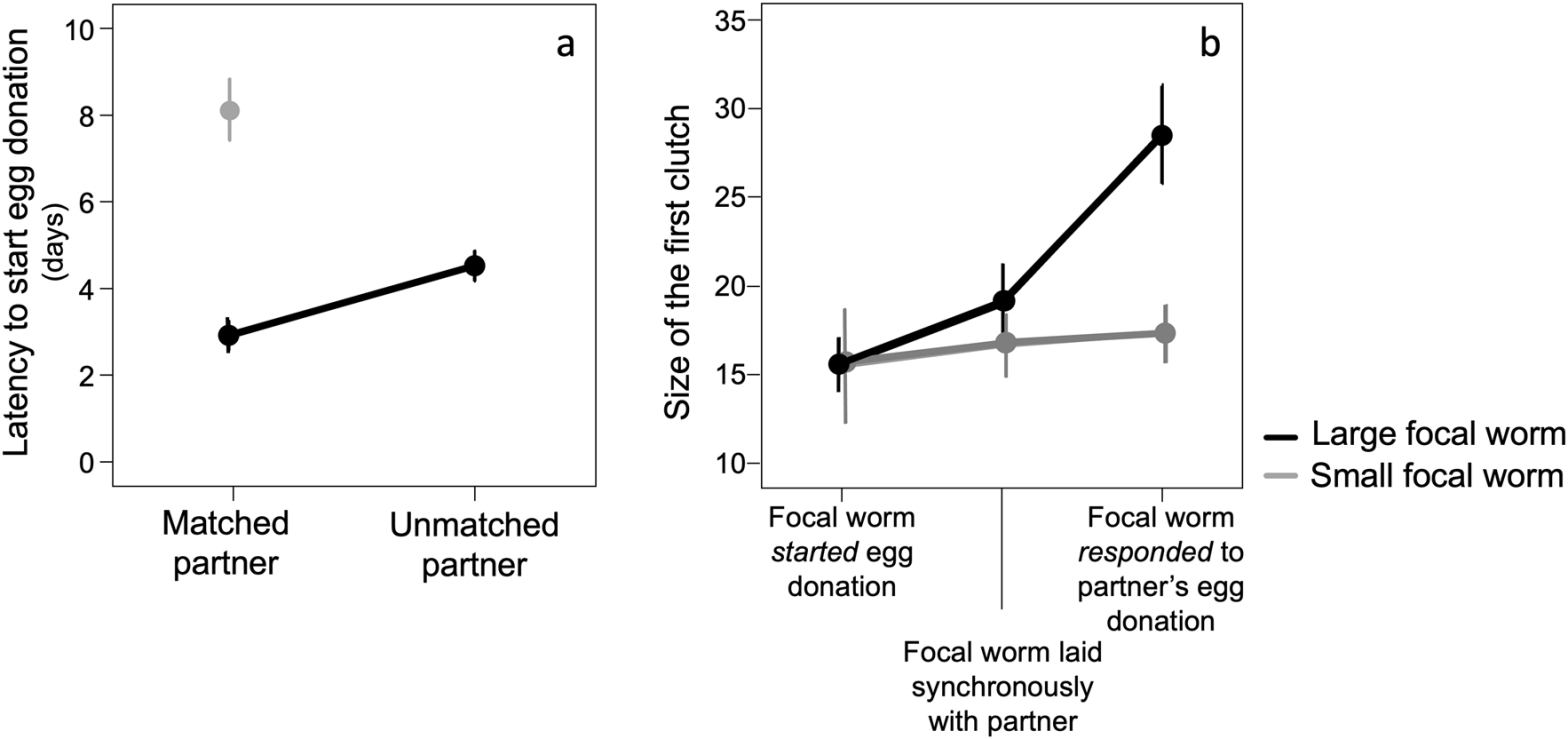
The start of reciprocity. (**a**) The latency to start egg donation for large and small focal worms as a function of the size of their partner (size-matched or size-unmatched partners). Large focal worms donated their eggs sooner than small focal worms, and significantly sooner when paired with large (matched) rather than small (unmatched) partners. (**b**) The size of the first clutch (the number of eggs) depended on the size of the focal worm (large or small) and the partner’s behavior (whether the focal worm *started* cooperation or *responded* to the partner’s donation). Large focal worms offered smaller clutches when they were the first to donate eggs than when they were reciprocating eggs to partners who had previously laid eggs. Small focal worms offered smaller, but relatively similarly sized egg-clutches, irrespective of condition.

Large focal worms started egg donation nearly 1.5 days earlier when paired with large rather than with small partners (2.92 days ± 0.336 *vs* 4.52 days ± 0.356, respectively). Large focal worms also started egg donation sooner than small focal worms (8.13 days ± 0.718) (Fig. 2a). Expectedly, no small worm laid before its large partner. The differences in latency to lay between large and small focal worms paired with matched or unmatched partners were highly significant (GLM; focal worm category [small]: (β = 1.025 ± 0.210, χ^2^ = 25.386, df = 1, P < 0.0001; partner’s size: [unmatched]: (β = 0.434 ± 0.192, χ^2^ = 5.625, df = 1, P = 0.018). Before the start of egg laying some worms were scored with intermediate levels of egg maturation (they partially resorbed yolk/eggs). However, the level of egg maturation entered in a preliminary model was non-significant, suggesting that the decision on whether to start egg donation was not primarily associated with egg-maturation level and therefore was removed from subsequent models.

#### The quality of first egg donation (or: start egg donation with caution, respond with confidence)

Focal worms also significantly adjusted the size of their donation to the behavior of their partners. The worms that *responded* to their partner’s egg donation laid clutches about twice as large than those that *started* egg donation (Fig. 2b). The worms that donated eggs synchronously with their partners laid clutches about 1/3 as large as those that started egg donation (Fig. 2b) (GLM, β [focal worm started egg donation] = − 0.374 ± 0.137; β [the two partners donated synchronously] = − 0.164 ± 0.139; χ^2^ = 7.535, df = 2, P = 0.023; as expected, large focal worms donated larger clutches than small worms: β [small focal worm] = − 0.250 ± 0.122, χ^2^ = 4.244, df = 1, P = 0.039; we retained the non-significant interaction between size of focal worm and the size of the partner because the AIC value was smaller than in the reduced model).

#### Reciprocity throughout the experiment

Once donation had started, worms donated proportionally more eggs to partners who donated more eggs; expectedly, the size of egg donation was also a function of their body size (GLMM, β [eggs donated by partners] = 0.260 ± 0.124, χ^2^ = 4.418, df = 1, P = 0.036; β [body size] = 0.337 ± 0.154, χ^2^ = 4.781, df = 1, P = 0.029), confirming previous findings^36^.

#### Investment in eggs throughout the experiment

**Figure 3:**
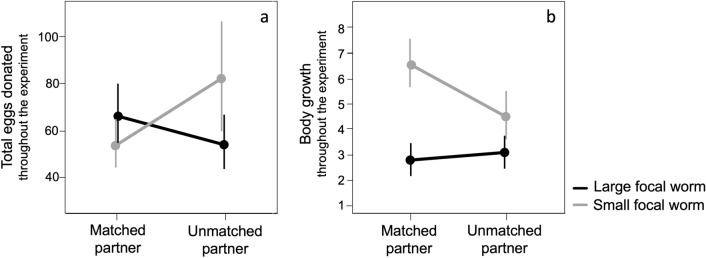
Egg investment and body growth throughout the experiment. (**a**) Egg investment (predicted values of the total number of eggs produced throughout the experiment) varied between large and small worms in different ways depending on the size of their partners. (**b**) The increase in body size (predicted number of segments gained throughout the experiment) varied between large and small worms in different ways depending on the size of their partners.

Over the experiment, focal worms donated different total numbers of eggs depending on whether they were large or small and paired with matched or unmatched partners (Fig. 3a). Overall, large worms paired with large partners donated *more eggs* compared to those paired with small partners; in contrast, small worms paired with small partners donated *fewer eggs* compared to those paired with large partners (GLM, size of focal worm*size of the partner: β [small worm*unmatched partner] = 0.633 ± 0.225, χ^2^ = 8.030, df = 1, P = 0.005; size of focal worm: β [small] = − 0.212 ± 0.117, χ^2^ = 3.325, df = 1, P = 0.0682; size of the partner: β [unmatched] = − 0.204 ± 0.111, χ^2^ = 3.377, df = 1, P = 0.066; controlling for the body size focal worms achieved at the end of the experiment: β [final body size] = 0.352 ± 0.058, χ^2^ = 45.576, df = 1, P < 0.0001) (Fig. 3a).

All worms increased their body size by 1–8 segments during the three-week experiment. However, large worms increased their size by 16.8% if paired with large partners and by 17.5% if paired with small partners. In contrast, small worms grew by 46.3% if paired with small partners but only by 29.3% if paired with large partners (Fig. 3b) (GLM, interaction size of focal worm*size of the partner: β [small worm*unmatched partner] = 0.465 ± 0.185, χ^2^ = 6.286, df = 1, P = 0.012; size of the focal worm: β [small] = 0.864 ± 0.129, χ^2^ = 47.743, df = 1, P < 0.0001; size of the partner: β [unmatched] = 0.088 ± 0.148, χ^2^ = 0.347, df = 1, P = 0.556; correcting for egg output, which increased with body size: β [number of eggs donated] = 0.352 ± 0.058, χ^2^ = 7.945, df = 1, P = 0.005).

### Experiment 2: behavioral interactions prior to reciprocity (dyads)

**Figure 4:**
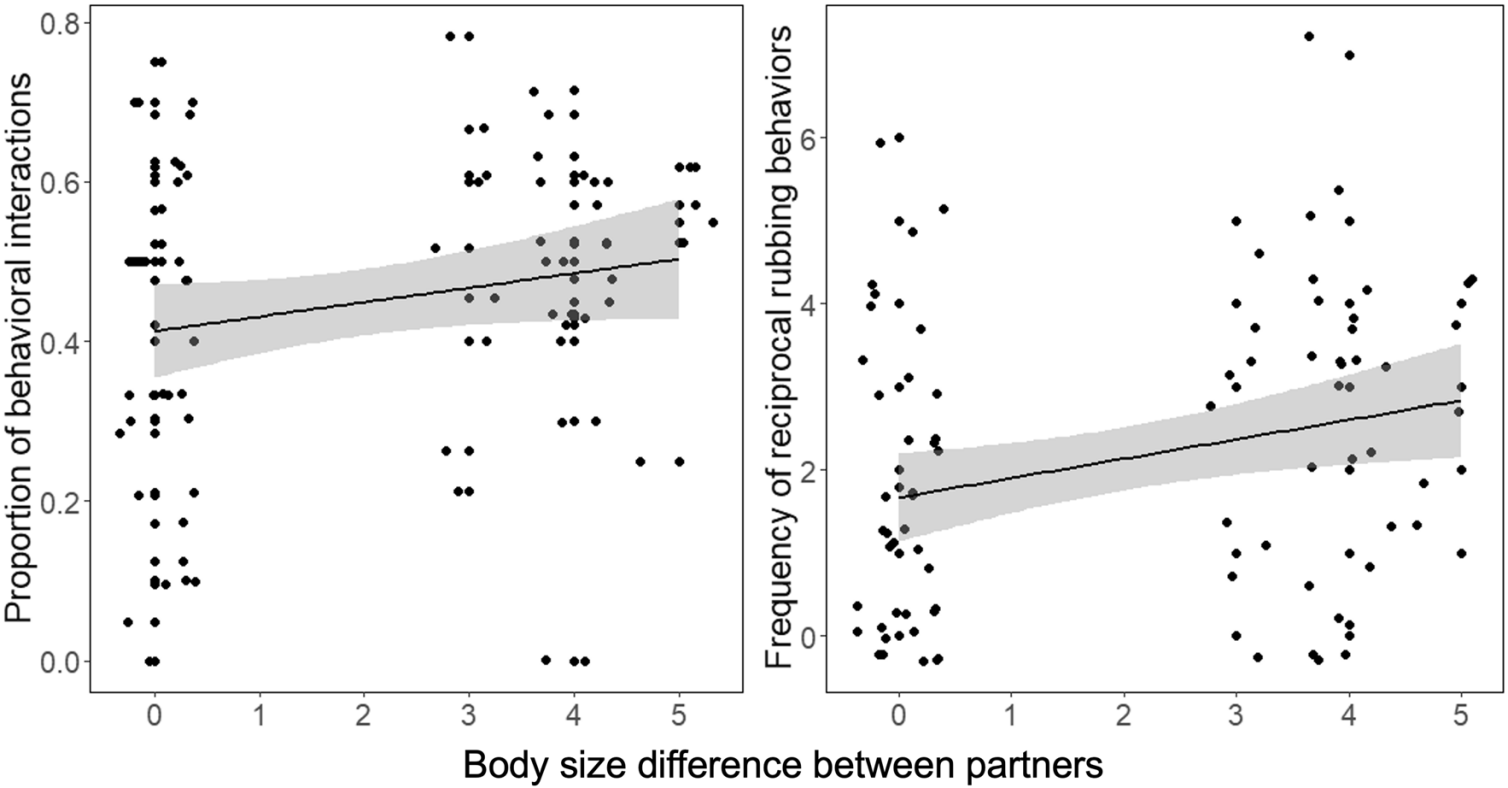
Behavioral interactions between partners. Both the proportion of behavioral interactions (**a**) and the frequency of reciprocal rubbing behaviors (**b**) increased with increasing size differences among partners (points jittered to prevent overlap).

We investigated whether newly paired partners interacted before starting egg exchanges and whether they regulated their interactions to suit their partner’s quality. Most worms began interacting within 30 min after pair formation. Behavioral interactions occurred significantly more often in more size-dissimilar pairs (Fig. 4) (GLM, (β [size difference between partners] = 0.061 ± 0.023, χ^2^ = 6.939, df = 1, P = 0.008. It’s worth noting that latency to laying (i.e., time worms spent together before laying eggs, which ranged 2–6 days) was entered in a preliminary model and had no significant effect on the frequency of behavioral interactions.

In particular, worms performed rubbing behaviors significantly more often with increasing size differences between partners (GLM, (β [body-size difference] = 0.092 ± 0.040, χ^2^ = 5.467, df = 1, P = 0.019; controlling for latency to laying: = 0.148 ± 0.073, χ^2^ = 3.930, df = 1, P = 0.047; and replicate: = 0.212 ± 0.075, χ^2^ = 8.112, df = 1, P = 0.004).

### Experiment 3: mate choice and partner quality (triplets)

**Figure 5:**
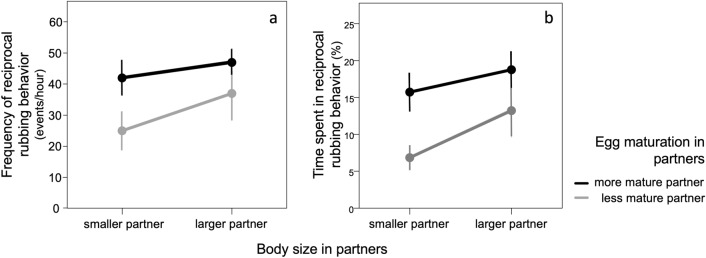
Reciprocal rubbing behavior when focal worms were given the choice between two partners of different quality: (**a**) frequency (events/hour) and (**b**) duration (proportion of time) of rubbing behavior as a function of the relative body size and egg maturation level of the two potential partners. When given such a choice, focal worms rubbed the larger and more mature worm more often and for longer.

When focal worms were offered the choice between two potential partners of different body size, the behavioral observations indicated that, prior to starting egg exchanges, focal worms rubbed the larger partner significantly more often and significantly longer than the smaller partner, especially if the larger partner was the more mature worm (Fig. 5) (LMM on rubbing frequency: β [size difference between potential partners] = 3.376 ± 1.504, χ^2^ = 5.037, df = 1, P = 0.025; β [less mature] = − 32.704 ± 10.589, χ^2^ = 9.539, df = 1, P = 0.002; β [size difference between focal worm and rubbing partner] = 2.691 ± 2.157, χ^2^ = 1.557, df = 1, P = 0.212; (removing the non-significant covariate “size difference between focal worm and rubbing partner” resulted in a larger AIC value); GLMM on rubbing duration: β [size difference between choice worm] = 0.227 ± 0.088, χ^2^ = 6.662, df = 1, P = 0.010; β [less mature] = − 2.296 ± 0.522, χ^2^ = 19.367, df = 1, P < 0.0001).

## Discussion

These results show that the quality of the partner as a reciprocator (and even its *expected* quality) affects the decision to start egg donations in egg-trading worms: large partners were offered larger clutches and received them sooner than small partners. Large worms also flexibly adjusted the size of their donation depending on whether they or their partner made the first move to start the sequence of mutual donations: when worms *started* the egg donation, they released relatively small egg clutches; when they *responded* to their partners’ first egg donation, they were significantly more generous and donated relatively large egg clutches. Overall, these results show that worms acted as prudent investors and provide support for the hypothesis that egg-trading in hermaphroditic worms conforms to the conditional reciprocity paradigm.

Picchi et al.^36^ showed that worms laid egg clutches conditional to receiving them and adjusted the size of their clutches to that received before (short-term contingency^7^). Here we show that worms also flexibly adjust their behavior in response to the *expected* action of their partner, as required for reciprocity to be stable^16,38^.

Our experimental design also provides one of the first test for the prediction that individuals involved in reciprocity “decrease aid to partners that are rendered unable to reciprocate”^13^. For instance, among birds, pied flycatchers assisted conspecific pairs in nest defense (mobbing) unless conspecific pairs had been previously experimentally prevented to help^58^. In an ingenious experiment on rats exposed to reciprocating and non-reciprocating partners on different days, subjects responded by reciprocating (food) only to previously reciprocating partners^10^. Reciprocity also regulates social interactions between dominants and subordinates in cooperative breeding fish. In the context of the pay-to-stay hypothesis, subordinate *Neoamprologus pulcher* individuals prevented from cooperating in shelter maintenance were tolerated less by dominants^59^. We did not manipulate worm ability to reciprocate but used “naturally” poor reciprocators (small worms) to test how high-quality reciprocators (large worms) interacted with them in a biologically meaningful setting: the circumstance where mate availability is low (rare species, sparse populations, limited mate-search efficiency^60^) and a mature hermaphrodite meets a low-quality partner. Small worms lay smaller egg clutches than large worms (fecundity correlates with body size^36,41^). Therefore, small worms offer fewer or no eggs in return and fail to pay back for the eggs received from large worms and are typically discarded as partners (^34^, this paper). However, a low-quality partner may be better than no partner at all, especially if mates are rarely encountered. When paired with small worms, large worms delayed the start of egg reciprocation, but then they did engage in repeated, although “prudent”, egg exchanges.

In our view, no hypothesis other than conditional reciprocity would predict that worms commensurate the start and maintenance of egg exchanges to their expected partner’s quality. Pseudo-reciprocity predicts that traits which confer fitness benefits to the actor while incidentally causing fitness benefits to the recipient may be selected^61–63^. Pseudo-reciprocity does not make any predictions related to a partner’s quality because it assumes that only one partner makes the investment^7^. This is not the case in the current investigation; the experiment on matched and unmatched partners documents that partner quality affects focal worms’ decisions. Large worms established pairwise reciprocal egg exchanges more promptly with large partners, which are more likely than small ones to pay back comparable returns at the next move. While exchanging eggs might be explained as pseudo-reciprocity, delaying egg-laying and releasing smaller egg-clutches to poor quality worms cannot.

One of the obstacles in applying the reciprocity paradigm to egg exchanges in hermaphrodites are the antithetical conditions which are thought to favor the evolution of egg trading and that of hermaphroditism^64,65^. Theoretical models predict that reciprocal egg-trading in hermaphrodites evolved under intermediate to high encounter rates and low sperm competition levels^64,66^, whereas hermaphroditism is considered stable when populations are sparse and consequently encounter rates are low^23,60^.

We have no evidence of the condition the worms experience in nature. They were rarely collected in the wild, which suggests—together with their small numbers at each collection—that they live in rare and sparse populations^67,68^, as it is typical of interstitial organisms^69^. They also produce aflagellate (immotile) sperm in very low numbers^70^, which implies that they have evolved under low sperm competition levels^71^. On the other hand, population density may increase locally and temporarily as worms intercept mucous trails, follow them and converge to relatively crowded spots^72^. Indeed, these worms can flexibly and rapidly adjust their sex allocation to mating opportunities and can compete for mating as males^49,53,54^, two traits which points to fluctuations in population density and mating opportunities^73^.

Although the worms in matched and unmatched pairs could not choose their partners, they modulated their egg donation behavior to their partner’s size in a way that suggests a preference for large partners. This was confirmed in the triadic experiment where worms preferentially rubbed the partner with the larger body size and the more mature eggs. Partner choice is one of the two mechanisms enforcing reciprocity according to biological market models^7,74,75^. By choosing partners which offer more profitable returns, players engaged in reciprocal exchanges ensure that “cheaters” (i.e., individuals that would not pay back the benefit received) get lower fitness gains than cooperators and enforce the stability of reciprocity^74,75^.

The focal worms’ preference for the larger and more mature partner in the triplet experiment suggests that worms follow a partner choice decision-making process based on a comparison of offers^74^, when given the possibility to do so. Large worms might have refrained from starting reciprocation with poor cooperators in anticipation of switching partners, should other partners show up. Indeed, partner switching emerges as the dominant behavioral response against non-reciprocating partners in individual-based simulations of evolving reciprocating populations, when tested against other behavioral mechanisms of partner control (i.e., punishing defecting partners and responding to defection with defection)^76^.

As partner switching was not an available option in our experimental conditions, large worms eventually donated eggs to their small partners, though they offered smaller clutches than to matched partners. In a previous experiment, pair bonds between reciprocating worms were more likely to break when an attractive worm (i.e., full of mature eggs) was introduced to reciprocating pairs^40^, supporting the hypothesis that worms assess the partners’ relative value by a comparison of offers. What do large worms gain by delaying the start of reciprocal exchanges when paired with unattractive partners? Worms might adjust their decisions based on both the risk that the partner will not return equitable egg donations and the cost/benefit ratio of current *vs* future mating probabilities (including egg senescence, see^65^. The debate on whether egg trading is in compliance with the reciprocity paradigm was grounded on the observation that cheaters, e.g., “fish which fertilize the eggs of the partner while keeping its own eggs as a bargaining chip to make the same arrangement with another fish”, would gain lower reproductive fitness than cooperative partners^77^. However, previous work documented that some mature hermaphrodites withhold their eggs and never lay them (and do fertilize partners’ eggs)^56^. These worms, which behave as “cheaters”, extend their lifespan by 70%, which supports the hypothesis that laying eggs is costly and that refraining from donating eggs may pay in terms of extended future mating opportunities^56^.

The ability to detect poor cooperators may appear as a complex one for organisms as simple-brained as annelid worms (although even organisms without nervous system, such as plants and fungi, detect partner investment and adjust their own investment accordingly^78^. Detecting fecundity (i.e., whether or not partners have mature eggs) is plausibly an ancestral skill used in the context of mate choice, a skill reported in aquatic annelids^79,80^ and mentioned as the potential first step in the evolution of egg trading^66^. But how do worms size up? Flatworms use a curious “sandwich posture” where two individuals flatten their body against each other, possibly to measure their partner’s body surface area^81^. Annelid body size is not fixed, and these worms can vary their body length and diameter because of their hydrostatic skeleton^82^. However, the prolonged and repeated bodily movements that worms exhibit while in physical contact prior to mating (e.g., rubbing^51^) might allow them to gather chemical and mechanosensory information on their partner’s body size, egg maturation and willingness to donate eggs. The fact that these behavioral interactions were more frequent between more size-dissimilar worms suggests that reaching a deal is more complex between more dissimilar worms and requires prolonged reciprocal interactions. Intriguingly, grooming interactions in vampire bats *Desmodus rotundus* predict future new food sharing events. Grooming has been interpreted as a way of testing the waters through low-cost behavioral physical exchanges before engaging into more costly moves, such as reciprocal food donations^57^. We do not know what information rubbing conveys in worms, but it is indeed a reciprocal behavior, to the point that it is difficult to identify which partner starts a rubbing bout (MCL, personal observation). Rubbing might be the worm equivalent of grooming in bats: a way of testing the waters through low-cost reciprocal physical interactions before investing in costly egg donations.

Small worms paired with large worms upregulated their egg investment at the expenses of body growth (tradeoff between reproductive investment and body growt^7,53,83^. This suggests a preference for large worms, confirmed by the current results on mate preferences in triplets. Large worms also exhibited a preference for large mates, as they started reciprocal egg exchanges more “optimistically” (i.e., sooner) when paired with matched partners. While this is a prediction of conditional reciprocity (matched partners are more likely to return goods of similar quality^38^), the correlation between phenotypic traits, and body size in particular, applies widely to animal mating pairs and is known as assortative mating^84,85^. Different hypotheses explain the widespread occurrence of positive assortative mating. For size assortment in particular, these include contingent conditions such as physical constraints on mating/copulation (e.g.,^81^) and spatial segregation (e.g., in hermaphroditic freshwater snails^86^; and fish^33^). In *O. diadema* worms, there is no physical limitation to mating between unmatched partners (fertilization is external although sperm are released inside the egg cocoon^39,51^), and the rarity of the species^39^ does not support spatial segregation in the wild. The current results suggest that size-assortative mating might enforce reciprocity and ensure matched benefits between partners, but the evolutionary link between assortative mating and egg trading deserves further theoretical investigation^66^. Partners with correlated quality are more likely to share both gains (reproductive success) and costs (resource investment in reproduction), eventually coupling their fitness across repeated pairwise reciprocal interactions^87^. In the real world, assortative mating would not always occur. In this case, testing the waters through behavioral interactions and prudent first moves may allow unmatched partners to build their reciprocal relationships through increasing investment and ensure that reciprocity is not invaded by non-reciprocators.

## Material and methods

### Rearing worms for the experiments

In lab cultures, two strains of worms exist which differ by a genetic marker^41,88^. Worms of one strain produce yellow eggs and those of the other produce white eggs. As the body walls are transparent, the worms appear as either yellow or white depending on the color of the eggs maturing in their body cavity. This allows individual recognition and the assignment of egg maternity/paternity in paired worms. The two strains have similar life history traits; however, white phenotype worms have lower fecundity, on average, than yellow-phenotype worms^36,43^. In the current experiments involving focal worms, all statistical comparisons were made between individuals which belonged to the same, yellow-phenotype strain.

For each experiment we generated a new cohort of worms from 40 to 80 pairs of either yellow- or white-phenotype parent worms which were moved from the mass cultures to separate bowls; when their progenies were newly sexually mature (i.e., same age of ~ 45 days) and virgin (i.e., no previous history of mating), they were used for the experiments. Worms were collected from several parental populations to avoid sibling pairings.

Experiments were carried out in 10 ml glass bowls where worms were kept in artificial sea water (35 ‰ salinity) in thermostatic cabinets at 20 °C and fed parboiled spinach *ad libitum*; water and food were renewed once a week. Observations and measures were performed with Leica EZ4 stereomicroscopes unless otherwise stated.

### Experimental settings

#### Experiment 1: reciprocation in matched and unmatched dyads

We designed a full factorial experiment to test whether pairwise reciprocal egg exchanges were adjusted to partner’s quality. We used body size as a proxy for quality because body size positively correlates with fecundity^36,41^. All worms entered the experiment when they had ready-to-lay eggs.

We set up 72 dyads each composed of a large yellow-phenotype worm (body size = 18 segments, thereafter, “large focal worm”) and a white-phenotype (~ albino) partner which was either size-matched (18 segments), or small (14 segments) (36 Large x Large dyads; 36 Large x Small dyads). To control for potential size-specific effects, we set up another 72 dyads where small yellow-phenotype worms (14 segments, thereafter, “small focal worms”) were paired with white-phenotype worms which were either size-matched (14 segments), or large (18 segments) (36 Small × Small dyads; 36 Small × Large dyads) (Fig. 1).

The 144 dyads were housed in separate bowls. During three weeks, we performed daily inspections of each bowl (7dd/7) and recorded the number of egg clutches, clutch size (number of eggs per clutch), egg color (yellow or white: matching egg and worm color allows for maternity assignment^88^) and worm body-size as the number of setigerous segments (body length is not a reliable measure of body size because annelid worms vary their body length due to their hydrostatic skeleton^82^). We also evaluated worms’ readiness to lay eggs by scoring their maturation as unripe, intermediate, or ready-to-lay eggs (eggs are visible through the transparent body wall). All worms had ready-to lay eggs at the start of the experiment, but their maturation-level changes as they lay, or as they refrain from laying and resorb part of the yolk. We removed egg clutches from the bowls once a week (i.e., before larvae hatched from their cocoon 8 days after egg laying^43^) to avoid changes in social condition (larvae have viable sperm as soon as they hatch and would have competed with mature hermaphrodites for egg fertilization^42,89^).

For statistical analyses, we calculated the latency to lay the first clutch by the focal worm as the time (days) from pairing to the laying of the first clutch by the focal worm. We used latency to lay the first clutch as a measure of the propensity of the focal worms to donate eggs. We used clutch size (the number of eggs in the clutch) as a measure of the quality of egg donation.

#### Experiment 2: behavioral interactions prior to reciprocity (dyads)

We investigated whether newly paired partners interacted before starting egg exchanges and whether they regulated their interactions to suit their partner’s quality. Previous work showed that mating (pseudocopulation, i.e., external egg fertilization which occurs inside the egg cocoon while partners are in physical contact with each other^39,51^) is preceded by partners following, being in contact and rubbing against each other’s bodies^51^. We hypothesized that worms in more size-dissimilar pairs would encounter broader conflicts over the start of reciprocal exchanges than those paired with size-matched partners and expected that they would interact more to solve the conflicts.

We set up 80 dyads, each composed of a yellow- and a white-phenotype worm (for logistic reasons, the experiment was performed by testing 20 dyads per week over four weeks). Within dyads worms had different body sizes (range: 16–22 segments; difference within dyads: 0–5 segments). Worms with similar levels of egg maturation were paired to minimize differences in physiological condition.

The dyads were housed in separate bowls. Behavioral observations began no sooner than 30 min after the experimental pairs were formed and were done using a magnifying glass to minimize worm disturbance (for the same reason bowls were kept out of the thermostatic cabinet, at room temperature, during behavioral observations). We recorded the frequency and quality of social interactions between partners at intervals of 30 min up to 15 times per day until one of the two worms laid the first cocoon, which occurred by 1–6 days (6–45 observations per pair, scan sampling). At each scan, we recorded whether the partners were rubbing, or following, or in physical contact with each other; “no interaction” was recorded when the worms were not interacting with each other. Each day, before starting behavioral observations, we inspected the bowls: the presence of cocoons at the start of the day signaled the end of the behavioral recording for that pair and was noted to calculate the latency to laying (e.g., time from pairing to the laying of the first clutch).

#### Experiment 3: mate choice and partner quality (triplets)

We tested whether worms exhibited a preference for the larger partners when given the choice between two worms. We set up 15 triplets of worms where one worm was the largest (hereafter “focal worm”; 19–26 segments and ready-to-lay eggs) and the other two (hereafter “choice worms”) differed from each other in body size (range of size difference: 0–7 segments; body size: 13–22 segments) but had similar physiological status (i.e., both had either ready-to lay eggs or no eggs). Each triplet was housed in the 9 mm-diameter lid of an Eppendorf Safe-Lock Tube 1.5 mL which fitted the field of view of the stereo microscope Leica M80, thus allowing continuous behavioral observations (preliminary analyses showed that triplets of worms live and lay eggs in such lids). We recorded the worms’ behavior for a total of 34 h (30 min per video, 1–4 videos per triplet) using the integrated digital camera Leica IC80 HD attached to the Leica M80 microscope (magnification 7.5). We used rubbing behavior as a measure of the focal worm preference: when rubbing, partners slide one against each other body in parallel and antiparallel directions for hours or days; rubbing is closely associated with mating (51). We measured frequency and duration of rubbing occurring between the focal worm and each of the two choice worms.

### Statistical analyses

All statistical analyses were performed in R (v. 0.99.896, http://www.r-project.org; with R studio, packages: ‘lme4’ and ‘stats’)^90^. Two-tailed p-values are reported.

For count data, we run models for Poisson distributed data (log link function), then checked for overdispersion. Where needed, we accounted for overdispersion by using a quasi-Poisson distribution family model in Generalized Linear Models (GLM) or by adding a case level random factor in Generalized Linear Mixed Effects Models (GLMM). We included the biologically reasonable interactions in the preliminary models and dropped them one by one, as well as factors and/or covariates, when they were non-significant; we compared the competing models’ AIC values and reported results from the models with the lowest AIC (Akaike Information Criterion) value to avoid information loss. Figures were done in R (package “interactions”) or in SPSS 22.0.

#### Experiment 1: reciprocation in matched and unmatched dyads

We tested whether the variation in latency to start egg donation by the focal worms depended on their size (two levels: large/small) and the size of the partner (two levels: matched/unmatched) using a GLM for Poisson distributed data. We included in the model the interaction between the two factors and controlled for the level of egg maturation. The dyads where worms laid the same day (n = 22) were excluded from the calculation.

To test whether focal worms adjusted the quality of their first clutch (number of eggs) to their partner’s behavior, we ran a GLM (quasi-Poisson distribution). We included in the model the size of the focal worm, the size of the partner (matched/unmatched) and a three-level factor which describes whether the focal worm laid its first egg clutch as the *start* of egg donation (first move), as the *response* to the partner’s egg donation (second move), or whether partners laid eggs synchronously (i.e., the same day). Latency to laying was added as a covariate to take into account that worms with a longer latency mature more eggs.

To test whether, across the experiment, egg donation by the focal worms was commensurate with that by their partners (i.e., whether reciprocity was adjusted to the partner’s response), we performed a GLMM (Poisson distribution) on the number of eggs donated by the focal worms at each egg-laying event and we entered the size of the partner (matched/unmatched), number of eggs donated by the partner and body size of the focal worm as independent variables. The identity of the focal worm and a case-level variable were included as random factors.

Finally, we reasoned that donating eggs implies investing resources in egg production, and we asked whether such egg investment depended on the relative partner size. We ran a GLM (quasi-Poisson distribution) on the production of eggs during the whole experiment (three weeks) where the size of the focal worm and the size of the partner (matched/unmatched) were factors (the body size that focal worms achieved at the end of the experiment was entered as a covariate to control for variation in fecundity due to size, after testing for collinearity). We also tested whether, in the hypothesis of a tradeoff between reproductive investment and body growth, egg production had an impact on body growth. We did that by running a GLM (Poisson distribution) on body growth (measured as the difference between body size at the start and at the end of the experiment) where the size of the focal worm and the size of the partner (matched/unmatched) were factors; their interaction was included to test for non-additive effects.

#### Experiment 2: behavioral interactions prior to reciprocity (dyads)

We tested whether behavioral interactions between partners varied in amount and/or quality as a function of their relative body size. Therefore, we ran a GLM (binomial family) to test whether the proportion of interactions (*vs* no interactions) was associated with body-size difference between partners, and ran a GLM (Poisson distribution) to test whether the frequency of rubbing, a typical reciprocal behavior that worms exhibit during courtship^65^, varied with body-size difference between partners. In both models, we controlled for the week the experiment was replicated (replicate), the size of the larger worm, its physiological condition and latency to lay (time from pairing to the laying of the first clutch; latency was set up at 3.5 in 26 out of 80 cases where we missed whether the worms laid eggs on the third or fourth day from pairing). Two dyads where no interaction was recorded were excluded from the analyses.

#### Experiment 3: mate choice and partner quality (triplets)

In the mate choice experiment, we tested whether focal worms varied their rubbing rate (number of rubbing/hour) and the proportion of time dedicated to rubbing (rubbing duration *vs* total observation time in sec) depending on the body-size difference between partners (body size of the worm rubbing with the focal worm minus body size of the third worm) (LMM on rubbing rate, GLMM for binomially distributed data on rubbing duration; triplet ID and video ID were random factors in both models).

### Ethic statement

The species used in the experiments (*O. diadema*) is not endangered or protected.
